# 9-(4-Meth­oxy­phen­yl)anthracene

**DOI:** 10.1107/S1600536812047149

**Published:** 2012-11-24

**Authors:** V. Silambarasan, T. Srinivasan, R. Sivasakthikumaran, A. K. Mohanakrishnan, D. Velmurugan

**Affiliations:** a CAS in Crystallography and Biophysics, University of Madras, Guindy Campus, Chennai-25, India; b Department of Organic Chemistry, University of Madras, Guindy Campus, Chennai-25, India

## Abstract

In the title compound, C_21_H_16_O, the dihedral angle between the anthracene ring system and the benzene ring is 74.3 (5)°. The anthracene ring system is essentially planar (r.m.s. deviation = 0.0257 Å) and the meth­oxy group lies in the plane of the benzene ring [C1—O1—C2—C7 torsion angle = 0.5 (2)°]. The crystal structure features π–π [centroid–centroid distance = 3.9487 (12) Å] and C–H⋯π inter­actions, forming a sheet running along the *a*-axis direction.

## Related literature

For applications of anthracene, see: Bae *et al.* (2010 ▶); Debbab *et al.* (2012 ▶). For a related structure, see: Wang *et al.* (2008 ▶).
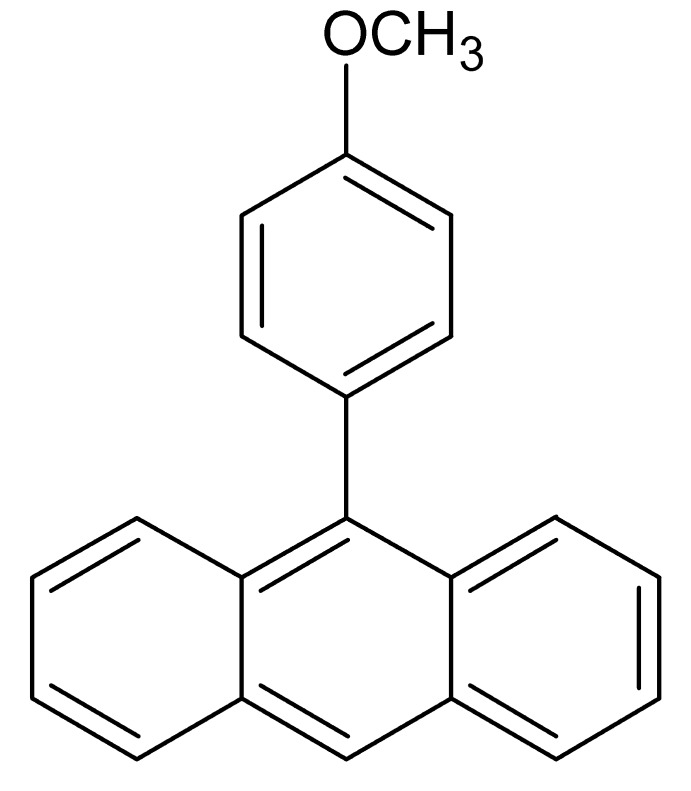


## Experimental

### 

#### Crystal data

C_21_H_16_O
*M* *_r_* = 284.34
Monoclinic, 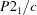

*a* = 13.5539 (5) Å
*b* = 15.0626 (5) Å
*c* = 7.6130 (2) Å
β = 99.219 (2)°
*V* = 1534.17 (9) Å^3^
*Z* = 4
Mo *K*α radiation
μ = 0.07 mm^−1^
*T* = 293 K
0.20 × 0.20 × 0.20 mm

#### Data collection

Bruker SMART APEXII area-detector diffractometer
Absorption correction: multi-scan (*SADABS*; Bruker, 2008 ▶) *T*_min_ = 0.981, *T*_max_ = 0.985
14917 measured reflections
3806 independent reflections
2317 reflections with *I* > 2σ(*I*)
*R*_int_ = 0.033

#### Refinement

*R*[*F* ^2^ > 2σ(*F* ^2^)] = 0.045
*wR*(*F* ^2^) = 0.130
*S* = 1.02
3806 reflections
201 parameters
H-atom parameters constrained
Δρ_max_ = 0.14 e Å^−3^
Δρ_min_ = −0.12 e Å^−3^

### 

Data collection: *APEX2* (Bruker, 2008 ▶); cell refinement: *SAINT* (Bruker, 2008 ▶); data reduction: *SAINT*; program(s) used to solve structure: *SHELXS97* (Sheldrick, 2008 ▶); program(s) used to refine structure: *SHELXL97* (Sheldrick, 2008 ▶); molecular graphics: *ORTEP-3* (Farrugia, 2012 ▶); software used to prepare material for publication: *SHELXL97* and *PLATON* (Spek, 2009 ▶).

## Supplementary Material

Crystal structure: contains datablock(s) global, I. DOI: 10.1107/S1600536812047149/pv2599sup1.cif

Structure factors: contains datablock(s) I. DOI: 10.1107/S1600536812047149/pv2599Isup2.hkl

Supplementary material file. DOI: 10.1107/S1600536812047149/pv2599Isup3.cml

Additional supplementary materials:  crystallographic information; 3D view; checkCIF report

## Figures and Tables

**Table 1:** Hydrogen-bond geometry (Å, °) *Cg*1 and *Cg*4 are the centroids of the C2–C7 and C16–C21 rings, respectively.

*D*—H⋯*A*	*D*—H	H⋯*A*	*D*⋯*A*	*D*—H⋯*A*
C7—H7⋯*Cg*4^i^	0.93	2.77	3.566 (2)	145
C11—H11⋯*Cg*1^ii^	0.93	2.87	3.724 (2)	154
C19—H19⋯*Cg*1^iii^	0.93	2.94	3.772 (2)	150
C21—H21⋯*Cg*4^iv^	0.93	2.88	3.711 (2)	150

Symmetry codes: (i) 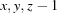; (ii) 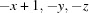; (iii) 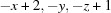; (iv) 
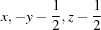
.

**Fig. 1.**
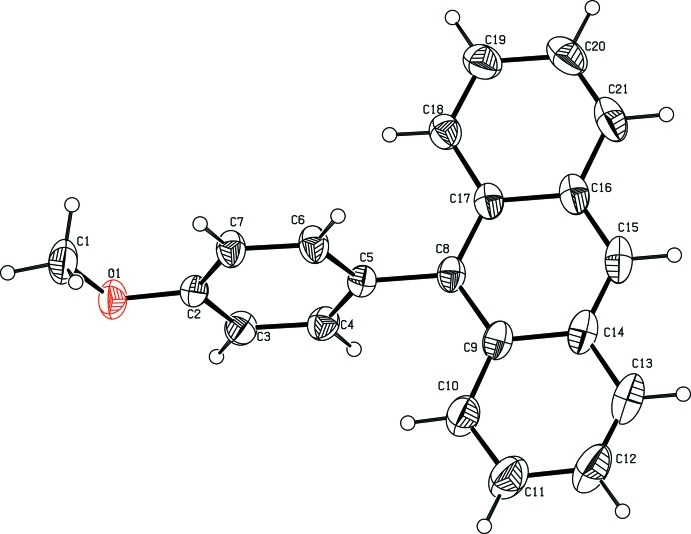
The molecular structure of the title compound with the atom numbering scheme. Displacement ellipsoids are drawn at the 30% probability level. H atoms are presented as small spheres of arbitrary radius.

**Fig. 2.**
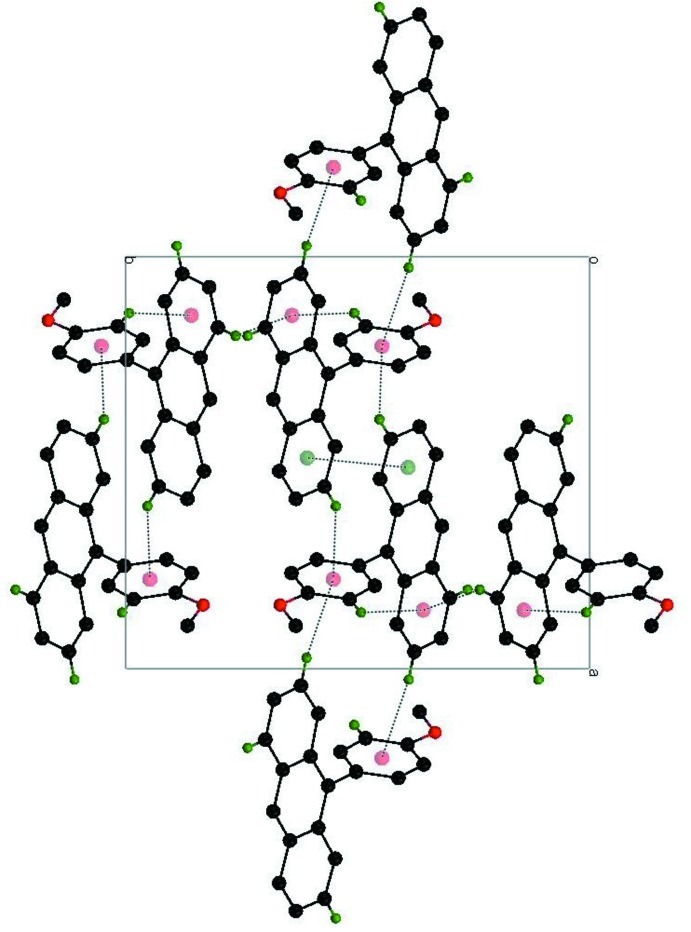
A view of the π···π and C—-H···π interactions (dotted lines) in the crystal structure of the title compound. H atoms non-participating in hydrogen-bonding were omitted for clarity.

## supplementary crystallographic information

### Comment

Anthracene is a solid polycyclic aromatic hydrocarbon consisting of three fused benzene rings. Its derivatives possess antimicrobial activity (Debbab *et al.*, 2012). It is used in the production of dyes and organic semiconductors (Bae *et al.*, 2010). In order to obtain detailed information on molecular conformations in the solid state, an X-ray crystallographic study of the title compound was carried out.

The anthracene ring system in the title molecule (Fig. 1) is essentially planar (rmsd = 0.0257 Å) and the methoxy group lies in the plane of the benzene ring with the torsion angle C1—O1—C2—C7 = 0.5 (2)°. The dihedral angle between the mean-planes of the anthracene and benzene rings is 74.3 (5)° showing that both the ring systems are almost perpendicular to each other. The bond lengths and angles in the title compound are comparable to those observed in a closely related compound (Wang *et al.*, 2008).

The π···π electron interaction is observed between the rings (C9—C14) [at *x*, *y*, *z*] and (C9—C14) [at 1 - *x*, - *y*, 1 - *z*] with the centroid-centroid distance 3.9487 (12) Å. In addition, the crystal packing is stabilized by C–H···π (Table. 1) types of interaction.

### Experimental

(4-Methoxyphenyl)(2-(phenyl(pivaloyloxy)methyl)phenyl)methyl)pivalate (0.5 g, 0.94 mmol) upon interaction with ZnBr_2_ (0.02 g, 0.13 mmol) followed by removal of solvent and column chromatographic purification (silica gel; hexane-ethyl acetate, 99:1) led to the isolation of product as a pale yellow solid (0.32 g, 87%). The compound was recrystalized from chloroform. Single crystals suitable for X-ray diffraction analysis were obtained by slow evaporation of a solution of the title compound in acetone at room temperature.

### Refinement

All H atoms were fixed geometrically and allowed to ride on their parent C atoms, with C—H distances 0.93 and 0.96 Å for aryl and methyl H-atoms with *U*_iso_(H) = 1.5*U*_eq_(methyl-C) and 1.2*U*_eq_(aryl-C).

### Crystal data

**Table:**

C_21_H_16_O	*F*(000) = 600
*M**_r_* = 284.34	*D*_x_ = 1.231 Mg m^−^^3^
Monoclinic, *P*2_1_/*c*	Mo *K*α radiation, λ = 0.71073 Å
Hall symbol: -P 2ybc	Cell parameters from 3806 reflections
*a* = 13.5539 (5) Å	θ = 2.0–28.3°
*b* = 15.0626 (5) Å	µ = 0.07 mm^−^^1^
*c* = 7.6130 (2) Å	*T* = 293 K
β = 99.219 (2)°	Block, colourless
*V* = 1534.17 (9) Å^3^	0.20 × 0.20 × 0.20 mm
*Z* = 4	

### Data collection

**Table:**

Bruker SMART APEXII area-detector diffractometer	3806 independent reflections
Radiation source: fine-focus sealed tube	2317 reflections with *I* > 2σ(*I*)
Graphite monochromator	*R*_int_ = 0.033
ω and φ scans	θ_max_ = 28.3°, θ_min_ = 2.0°
Absorption correction: multi-scan (*SADABS*; Bruker, 2008)	*h* = −18→16
*T*_min_ = 0.981, *T*_max_ = 0.985	*k* = −19→19
14917 measured reflections	*l* = −10→10

### Refinement

**Table:**

Refinement on *F*^2^	Secondary atom site location: difference Fourier map
Least-squares matrix: full	Hydrogen site location: inferred from neighbouring sites
*R*[*F*^2^ > 2σ(*F*^2^)] = 0.045	H-atom parameters constrained
*wR*(*F*^2^) = 0.130	*w* = 1/[σ^2^(*F*_o_^2^) + (0.0556*P*)^2^ + 0.1555*P*] where *P* = (*F*_o_^2^ + 2*F*_c_^2^)/3
*S* = 1.02	(Δ/σ)_max_ < 0.001
3806 reflections	Δρ_max_ = 0.14 e Å^−^^3^
201 parameters	Δρ_min_ = −0.12 e Å^−^^3^
0 restraints	Extinction correction: *SHELXL*, Fc^*^=kFc[1+0.001xFc^2^λ^3^/sin(2θ)]^-1/4^
Primary atom site location: structure-invariant direct methods	Extinction coefficient: 0.019 (2)

### Special details

**Table:**

Geometry. All e.s.d.'s (except the e.s.d. in the dihedral angle between two l.s. planes) are estimated using the full covariance matrix. The cell e.s.d.'s are taken into account individually in the estimation of e.s.d.'s in distances, angles and torsion angles; correlations between e.s.d.'s in cell parameters are only used when they are defined by crystal symmetry. An approximate (isotropic) treatment of cell e.s.d.'s is used for estimating e.s.d.'s involving l.s. planes.
Refinement. Refinement of *F*^2^ against ALL reflections. The weighted *R*-factor *wR* and goodness of fit *S* are based on *F*^2^, conventional *R*-factors *R* are based on *F*, with *F* set to zero for negative *F*^2^. The threshold expression of *F*^2^ > σ(*F*^2^) is used only for calculating *R*-factors(gt) *etc*. and is not relevant to the choice of reflections for refinement. *R*-factors based on *F*^2^ are statistically about twice as large as those based on *F*, and *R*- factors based on ALL data will be even larger.

### Fractional atomic coordinates and isotropic or equivalent isotropic displacement parameters (Å^2^)

**Table:**

	*x*	*y*	*z*	*U*_iso_*/*U*_eq_	
O1	0.84535 (8)	−0.16715 (6)	−0.12133 (14)	0.0625 (3)	
C5	0.75001 (9)	0.00335 (9)	0.24854 (16)	0.0444 (3)	
C8	0.71622 (11)	0.06367 (9)	0.38283 (16)	0.0469 (3)	
C4	0.73498 (10)	−0.08803 (9)	0.25059 (19)	0.0513 (4)	
H4	0.7022	−0.1127	0.3373	0.062*	
C6	0.79836 (11)	0.03687 (9)	0.11597 (18)	0.0543 (4)	
H6	0.8091	0.0978	0.1112	0.065*	
C2	0.81595 (9)	−0.10761 (9)	−0.00379 (17)	0.0471 (3)	
C17	0.78712 (11)	0.10166 (9)	0.51685 (17)	0.0484 (3)	
C9	0.61419 (11)	0.08466 (10)	0.37219 (18)	0.0542 (4)	
C3	0.76772 (11)	−0.14246 (9)	0.12656 (19)	0.0526 (4)	
H3	0.7572	−0.2034	0.1309	0.063*	
C16	0.75482 (13)	0.16124 (9)	0.64257 (18)	0.0571 (4)	
C18	0.89164 (11)	0.08456 (10)	0.53287 (18)	0.0553 (4)	
H18	0.9144	0.0464	0.4521	0.066*	
C14	0.58323 (13)	0.14597 (11)	0.4959 (2)	0.0633 (4)	
C7	0.83127 (11)	−0.01703 (9)	−0.00955 (18)	0.0535 (4)	
H7	0.8635	0.0074	−0.0972	0.064*	
C19	0.95862 (14)	0.12230 (11)	0.6623 (2)	0.0687 (5)	
H19	1.0264	0.1101	0.6690	0.082*	
C15	0.65393 (14)	0.18180 (10)	0.6282 (2)	0.0681 (5)	
H15	0.6333	0.2208	0.7099	0.082*	
C21	0.82821 (16)	0.19878 (11)	0.7773 (2)	0.0730 (5)	
H21	0.8080	0.2371	0.8605	0.088*	
C10	0.53910 (12)	0.04832 (12)	0.2391 (2)	0.0710 (5)	
H10	0.5573	0.0077	0.1581	0.085*	
C20	0.92599 (16)	0.18010 (12)	0.7871 (2)	0.0772 (5)	
H20	0.9723	0.2054	0.8765	0.093*	
C1	0.89552 (14)	−0.13330 (12)	−0.2567 (2)	0.0788 (5)	
H1A	0.8533	−0.0912	−0.3275	0.118*	
H1B	0.9110	−0.1812	−0.3309	0.118*	
H1C	0.9562	−0.1046	−0.2035	0.118*	
C13	0.47972 (16)	0.16896 (14)	0.4771 (3)	0.0847 (6)	
H13	0.4587	0.2095	0.5555	0.102*	
C11	0.44201 (14)	0.07163 (15)	0.2281 (3)	0.0893 (6)	
H11	0.3945	0.0468	0.1402	0.107*	
C12	0.41231 (16)	0.13311 (17)	0.3484 (3)	0.0960 (7)	
H12	0.3454	0.1491	0.3386	0.115*	

### Atomic displacement parameters (Å^2^)

**Table:**

	*U*^11^	*U*^22^	*U*^33^	*U*^12^	*U*^13^	*U*^23^
O1	0.0721 (7)	0.0500 (6)	0.0713 (7)	0.0036 (5)	0.0293 (6)	−0.0138 (5)
C5	0.0457 (7)	0.0464 (7)	0.0423 (6)	0.0033 (6)	0.0101 (6)	−0.0003 (6)
C8	0.0587 (9)	0.0420 (7)	0.0433 (7)	0.0043 (6)	0.0183 (6)	0.0026 (6)
C4	0.0539 (8)	0.0488 (8)	0.0546 (8)	−0.0021 (6)	0.0190 (7)	0.0041 (6)
C6	0.0739 (10)	0.0397 (7)	0.0537 (8)	−0.0032 (7)	0.0235 (7)	−0.0027 (6)
C2	0.0444 (7)	0.0456 (7)	0.0526 (7)	0.0043 (6)	0.0112 (6)	−0.0073 (6)
C17	0.0664 (9)	0.0384 (7)	0.0439 (7)	−0.0020 (6)	0.0198 (6)	0.0027 (6)
C9	0.0628 (9)	0.0552 (9)	0.0485 (7)	0.0108 (7)	0.0207 (7)	0.0060 (7)
C3	0.0572 (9)	0.0384 (7)	0.0645 (9)	−0.0013 (6)	0.0169 (7)	−0.0005 (6)
C16	0.0865 (11)	0.0409 (7)	0.0494 (8)	−0.0037 (7)	0.0269 (8)	−0.0005 (6)
C18	0.0656 (10)	0.0521 (8)	0.0504 (8)	−0.0068 (7)	0.0163 (7)	−0.0022 (7)
C14	0.0776 (11)	0.0613 (10)	0.0580 (9)	0.0177 (8)	0.0322 (8)	0.0073 (8)
C7	0.0692 (10)	0.0459 (8)	0.0507 (7)	−0.0032 (7)	0.0258 (7)	−0.0015 (6)
C19	0.0736 (11)	0.0719 (11)	0.0605 (9)	−0.0202 (9)	0.0111 (8)	−0.0043 (8)
C15	0.1015 (14)	0.0520 (9)	0.0599 (9)	0.0122 (9)	0.0405 (9)	−0.0029 (7)
C21	0.1192 (16)	0.0489 (9)	0.0561 (9)	−0.0182 (10)	0.0303 (10)	−0.0120 (7)
C10	0.0626 (10)	0.0904 (13)	0.0613 (9)	0.0193 (9)	0.0136 (8)	−0.0032 (9)
C20	0.1028 (15)	0.0692 (11)	0.0601 (10)	−0.0349 (11)	0.0147 (10)	−0.0097 (8)
C1	0.0935 (13)	0.0728 (12)	0.0811 (11)	−0.0002 (10)	0.0480 (10)	−0.0192 (10)
C13	0.0924 (14)	0.0910 (14)	0.0807 (12)	0.0338 (11)	0.0441 (11)	0.0065 (11)
C11	0.0623 (11)	0.1274 (18)	0.0782 (11)	0.0241 (11)	0.0109 (9)	−0.0017 (12)
C12	0.0735 (13)	0.1313 (19)	0.0882 (14)	0.0398 (13)	0.0287 (12)	0.0091 (14)

### Geometric parameters (Å, º)

**Table:**

O1—C2	1.3704 (15)	C18—H18	0.9300
O1—C1	1.4176 (18)	C14—C15	1.384 (2)
C5—C6	1.3838 (17)	C14—C13	1.430 (3)
C5—C4	1.3919 (18)	C7—H7	0.9300
C5—C8	1.4935 (17)	C19—C20	1.411 (2)
C8—C17	1.4063 (19)	C19—H19	0.9300
C8—C9	1.408 (2)	C15—H15	0.9300
C4—C3	1.3766 (19)	C21—C20	1.345 (3)
C4—H4	0.9300	C21—H21	0.9300
C6—C7	1.3813 (18)	C10—C11	1.352 (2)
C6—H6	0.9300	C10—H10	0.9300
C2—C3	1.3768 (18)	C20—H20	0.9300
C2—C7	1.3818 (19)	C1—H1A	0.9600
C17—C18	1.426 (2)	C1—H1B	0.9600
C17—C16	1.4309 (19)	C1—H1C	0.9600
C9—C10	1.425 (2)	C13—C12	1.341 (3)
C9—C14	1.429 (2)	C13—H13	0.9300
C3—H3	0.9300	C11—C12	1.406 (3)
C16—C15	1.389 (2)	C11—H11	0.9300
C16—C21	1.426 (2)	C12—H12	0.9300
C18—C19	1.353 (2)		
			
C2—O1—C1	117.58 (11)	C6—C7—C2	119.44 (12)
C6—C5—C4	117.14 (12)	C6—C7—H7	120.3
C6—C5—C8	120.68 (12)	C2—C7—H7	120.3
C4—C5—C8	122.17 (11)	C18—C19—C20	120.22 (17)
C17—C8—C9	120.07 (12)	C18—C19—H19	119.9
C17—C8—C5	119.69 (12)	C20—C19—H19	119.9
C9—C8—C5	120.21 (13)	C14—C15—C16	121.90 (14)
C3—C4—C5	121.16 (12)	C14—C15—H15	119.1
C3—C4—H4	119.4	C16—C15—H15	119.1
C5—C4—H4	119.4	C20—C21—C16	121.62 (16)
C7—C6—C5	122.23 (13)	C20—C21—H21	119.2
C7—C6—H6	118.9	C16—C21—H21	119.2
C5—C6—H6	118.9	C11—C10—C9	121.45 (17)
O1—C2—C3	116.26 (12)	C11—C10—H10	119.3
O1—C2—C7	124.36 (12)	C9—C10—H10	119.3
C3—C2—C7	119.38 (12)	C21—C20—C19	120.27 (16)
C8—C17—C18	122.87 (12)	C21—C20—H20	119.9
C8—C17—C16	119.58 (13)	C19—C20—H20	119.9
C18—C17—C16	117.54 (13)	O1—C1—H1A	109.5
C8—C9—C10	122.52 (13)	O1—C1—H1B	109.5
C8—C9—C14	119.77 (14)	H1A—C1—H1B	109.5
C10—C9—C14	117.70 (14)	O1—C1—H1C	109.5
C4—C3—C2	120.65 (13)	H1A—C1—H1C	109.5
C4—C3—H3	119.7	H1B—C1—H1C	109.5
C2—C3—H3	119.7	C12—C13—C14	121.20 (17)
C15—C16—C21	122.17 (15)	C12—C13—H13	119.4
C15—C16—C17	119.34 (15)	C14—C13—H13	119.4
C21—C16—C17	118.48 (15)	C10—C11—C12	120.5 (2)
C19—C18—C17	121.86 (14)	C10—C11—H11	119.7
C19—C18—H18	119.1	C12—C11—H11	119.7
C17—C18—H18	119.1	C13—C12—C11	120.56 (18)
C15—C14—C9	119.31 (14)	C13—C12—H12	119.7
C15—C14—C13	122.16 (16)	C11—C12—H12	119.7
C9—C14—C13	118.53 (17)		
			
C6—C5—C8—C17	−72.83 (17)	C8—C17—C18—C19	179.58 (13)
C4—C5—C8—C17	106.98 (15)	C16—C17—C18—C19	0.5 (2)
C6—C5—C8—C9	105.19 (16)	C8—C9—C14—C15	−2.0 (2)
C4—C5—C8—C9	−74.99 (17)	C10—C9—C14—C15	179.23 (14)
C6—C5—C4—C3	0.5 (2)	C8—C9—C14—C13	177.33 (14)
C8—C5—C4—C3	−179.30 (13)	C10—C9—C14—C13	−1.4 (2)
C4—C5—C6—C7	−0.3 (2)	C5—C6—C7—C2	−0.1 (2)
C8—C5—C6—C7	179.51 (13)	O1—C2—C7—C6	179.96 (13)
C1—O1—C2—C3	−179.79 (13)	C3—C2—C7—C6	0.3 (2)
C1—O1—C2—C7	0.5 (2)	C17—C18—C19—C20	0.3 (2)
C9—C8—C17—C18	−178.74 (12)	C9—C14—C15—C16	1.3 (2)
C5—C8—C17—C18	−0.71 (19)	C13—C14—C15—C16	−178.05 (15)
C9—C8—C17—C16	0.35 (19)	C21—C16—C15—C14	179.21 (14)
C5—C8—C17—C16	178.38 (11)	C17—C16—C15—C14	0.3 (2)
C17—C8—C9—C10	179.89 (13)	C15—C16—C21—C20	−178.28 (15)
C5—C8—C9—C10	1.9 (2)	C17—C16—C21—C20	0.7 (2)
C17—C8—C9—C14	1.2 (2)	C8—C9—C10—C11	−177.86 (16)
C5—C8—C9—C14	−176.82 (12)	C14—C9—C10—C11	0.9 (2)
C5—C4—C3—C2	−0.3 (2)	C16—C21—C20—C19	0.1 (3)
O1—C2—C3—C4	−179.78 (13)	C18—C19—C20—C21	−0.6 (2)
C7—C2—C3—C4	−0.1 (2)	C15—C14—C13—C12	−179.66 (18)
C8—C17—C16—C15	−1.1 (2)	C9—C14—C13—C12	1.0 (3)
C18—C17—C16—C15	178.03 (13)	C9—C10—C11—C12	0.2 (3)
C8—C17—C16—C21	179.92 (12)	C14—C13—C12—C11	0.0 (3)
C18—C17—C16—C21	−0.94 (19)	C10—C11—C12—C13	−0.6 (3)

### Hydrogen-bond geometry (Å, º)

Cg1 and Cg4 are the centroids of the C2–C7 and C16–C21 rings, respectively.

**Table:**

*D*—H···*A*	*D*—H	H···*A*	*D*···*A*	*D*—H···*A*
C7—H7···*Cg*4^i^	0.93	2.77	3.566 (2)	145
C11—H11···*Cg*1^ii^	0.93	2.87	3.724 (2)	154
C19—H19···*Cg*1^iii^	0.93	2.94	3.772 (2)	150
C21—H21···*Cg*4^iv^	0.93	2.88	3.711 (2)	150

Symmetry codes: (i) *x*, *y*, *z*−1; (ii) −*x*+1, −*y*, −*z*; (iii) −*x*+2, −*y*, −*z*+1; (iv) *x*, −*y*−1/2, *z*−1/2.

## References

[bb1] Bae, S. Y., Jung, K. H., Hoang, M. H., Kim, K. H., Lee, T. W., Cho, M. J., Jin, J., Lee, D. H., Chung, D. S., Park, C. E. & Choi, D. H. (2010). *Synth. Met.* **160**, 1022–1029.

[bb2] Bruker (2008). *APEX2, *SAINT** and *SADABS* Bruker AXS Inc., Madison, Wisconsin, USA.

[bb3] Debbab, A., Aly, A. H., Edrada-Ebel, R., Wray, V., Pretsch, A., Pescitelli, G., Kurtan, T. & Proksch, P. (2012). *Eur. J. Org. Chem.* pp. 1351–1359.

[bb4] Farrugia, L. J. (2012). *J. Appl. Cryst.* **45**, 849–854.

[bb5] Sheldrick, G. M. (2008). *Acta Cryst.* A**64**, 112–122. 10.1107/S0108767307043930 18156677

[bb6] Spek, A. L. (2009). *Acta Cryst.* D**65**, 148–155. 10.1107/S090744490804362X PMC2631630 19171970

[bb7] Wang, L., You, W., Huang, W., Jiang, J.-C. & Yao, C. (2008). *Acta Cryst.* E**64**, o487. 10.1107/S1600536808001669 PMC2960177 21201510

